# Accuracy and Repeatability of ChatGPT Based on a Set of Multiple-Choice Questions on Objective Tests of Hearing

**DOI:** 10.7759/cureus.59857

**Published:** 2024-05-08

**Authors:** Krzysztof Kochanek, Henryk Skarzynski, Wiktor W Jedrzejczak

**Affiliations:** 1 Department of Experimental Audiology, Institute of Physiology and Pathology of Hearing, Warsaw, POL; 2 Otorhinolaryngosurgery Clinic, Institute of Physiology and Pathology of Hearing, Warsaw, POL

**Keywords:** audiology, clinical electrophysiology, hearing assessment, consistency, large language model, repeatability, chatgpt

## Abstract

Introduction: ChatGPT has been tested in many disciplines, but only a few have involved hearing diagnosis and none to physiology or audiology more generally. The consistency of the chatbot’s responses to the same question posed multiple times has not been well investigated either. This study aimed to assess the accuracy and repeatability of ChatGPT 3.5 and 4 on test questions concerning objective measures of hearing. Of particular interest was the short-term repeatability of responses which was here tested on four separate days extended over one week.

Methods: We used 30 single-answer, multiple-choice exam questions from a one-year course on objective methods of testing hearing. The questions were posed five times to both ChatGPT 3.5 (the free version) and ChatGPT 4 (the paid version) on each of four days (two days one week and two days the following week). The accuracy of the responses was evaluated in terms of a response key. To evaluate the repeatability of the responses over time, percent agreement and Cohen’s Kappa were calculated.

Results: The overall accuracy of ChatGPT 3.5 was 48-49%, while that of ChatGPT 4 was 65-69%. ChatGPT 3.5 consistently failed to pass the threshold of 50% correct responses. Within a single day, the percent agreement was 76-79% for ChatGPT 3.5 and 87-88% for ChatGPT 4 (Cohen’s Kappa 0.67-0.71 and 0.81-0.84 respectively). The percent agreement between responses from different days was 75-79% for ChatGPT 3.5 and 85-88% for ChatGPT 4 (Cohen’s Kappa 0.65-0.69 and 0.80-0.85 respectively).

Conclusion: ChatGPT 4 outperforms ChatGPT 3.5 both in accuracy and higher repeatability over time. However, the great variability of the responses casts doubt on possible professional applications of both versions.

## Introduction

Chatbots, conversational systems based on large language models (LLMs), have recently revolutionized both public and professional spheres across various disciplines [1,2]. Efforts to explore their applications are growing, with significant interest in sectors such as healthcare - where chatbots are being tested as patient support tools and as educational aids in scenario-based training [3,4]. Among these chatbots, ChatGPT, developed by OpenAI (San Francisco, CA, USA), is perhaps the most well known, prompting much interest in chatbot technology [5].

Presently, two versions of ChatGPT are accessible to the general public: the freely available ChatGPT 3.5, based on an earlier LLM, and the more advanced, subscription-based ChatGPT 4. Research has shown that ChatGPT 4 outperforms its predecessor, demonstrating superior accuracy in various fields such as dermatology, where it achieved 80-85% accuracy compared to 60-70% for ChatGPT 3.5 [6]. Similar improvements are observed in orthopedic assessments and in general medical examinations, with ChatGPT 4 consistently outperforming ChatGPT 3.5 [7,8].

Despite broad testing across scientific and medical fields, audiology remains underexplored. A search on PubMed for "chatgpt [Title/Abstract] AND audiology [Title/Abstract]" returned no results, compared with 35 papers found for otolaryngology and even more in fields like dermatology and ophthalmology (as of April 5, 2024). Preliminary studies in audiology suggest that while ChatGPT, alongside other chatbots like Google Bard (now Gemini) and Bing Chat (now Copilot), shows promise, it also exhibits errors and inaccuracies that underscore the need for careful oversight when used in specialized fields [9]. This is particularly evident in some audiology subtopics such as tinnitus, where the responses, although quite impressive, occasionally stray from the topic and, crucially, totally lack citations [10]. These latter two studies suggest that ChatGPT has the potential to provide information in more specialized medical fields like audiology and in specific topics like tinnitus, but still requires improvement before being reliable enough for serious applications.

While the correctness of ChatGPT’s responses seems quite well researched, there seems to be little information on test-retest repeatability, with only a few studies on that aspect available so far. For example, in one study exploring how ChatGPT might be used to create norming data, the reliability was about 85% when results from different days were compared [11]. Similarly, another study on ChatGPT’s performance in prosthodontics showed a repeatability of 88% [12]. However, it is unlikely that results from dentistry provide a good estimate of reliability in audiology or otorhinolaryngology.

This study aims to fill these gaps by assessing the accuracy and repeatability of ChatGPT versions 3.5 and 4 in answering test questions about the physiology of hearing. Objective measures such as tympanometry [13], middle ear muscle response [14], otoacoustic emissions [15], and auditory brainstem responses [16] are paramount in audiology and here they served as the basis for our investigation. By focusing on the short-term repeatability of responses - within a single day, across two days, and over a week - this study sought to determine the feasibility of deploying ChatGPT in a clinical audiology setting, evaluating its potential as a reliable diagnostic or educational aid.

## Materials and methods

The responses of two versions of OpenAI’s chatbot ChatGPT to a set of single-choice questions were evaluated. The versions were ChatGPT 3.5 (free) and ChatGPT 4 (paid), with version 4 being the more advanced. The knowledge cutoff for ChatGPT 3.5 was early 2021, meaning it was last trained on new data around that time. ChatGPT 4, on the other hand, has a knowledge cutoff of April 2023. In practical terms, the cutoffs represent the most recent data included in the training set before the model was finalized. While version 3.5 is free, version 4 requires a subscription (a monthly cost of 24.60 USD at the time we tested it).

We used a set of 30 single-answer, multiple-choice questions developed by one of the authors (KK). The questions were selected from exams given to students undertaking a one-year course on objective hearing testing which is conducted at the Institute of Physiology and Pathology of Hearing, Poland, over the last 20 years. The questions relate to physiological measurements of hearing status, in particular tympanometry, middle ear muscle responses, otoacoustic emissions, and auditory brainstem responses. The questions are based on common audiological knowledge described in research papers as well as in books (both in English (e.g. [17]) and our native Polish (e.g. [18])). We did not use questions from any published book as copyright makes it difficult to publish the questions and the key. All questions had a choice of four answers from which one was correct. Table 1 presents examples of two questions, while all the questions together with the key are provided in the Appendices. Although our native language is Polish we presented the questions to the chatbots in English translation in order to make it international.

**Table 1:** Examples of questions (from a total of 30) used in the testing. All questions, together with the answer key, can be found in the Appendices.

Question number	Question
3	The frequency of the measuring tone for tympanometry in a child aged 3 months should be: (a) 220 Hz (b) 1000 Hz (c) 50 Hz (d) 226 Hz
30	Auditory brainstem responses have the following clinical applications: (a) for newborn hearing screening (b) for hearing threshold testing (c) for hearing threshold testing, newborn hearing screening, and differential diagnosis of hearing disorders (d) only for the diagnosis of retrocochlear disorders

All questions were presented to both chatbots on four specific days. First on two consecutive days (01-02.04.2024) and then two consecutive days a week later (08-09.04.2024). The set of questions was presented five times each day (that is, in total 20 tests were given for each version of ChatGPT). Each day’s trials were made with only a gap of about five minutes between them (after resetting the conversation). The responses of the chatbots are provided in the Supplementary file.

All analyses were made in Matlab (version 2023b, MathWorks, Natick, MA, USA). Percent agreement and Cohen’s Kappa [19] were used to evaluate agreement, correctness of responses, and test-retest repeatability. We used both numerical measures as percent agreement is more accessible to the common reader whereas Cohen’s Kappa has the advantage that it also tests for the possibility of agreement occurring by chance. The values of percent agreement and Kappa can be interpreted as <0.0, poor; 0.0-0.2, slight; 0.2-0.4, fair; 0.4-0.6, moderate; 0.6-0.8, substantial; and 0.8-1.0, almost perfect agreement [20]. Repeated measures analysis of variance (rmANOVA) was used to assess the effect of testing at different moments. For pairwise comparisons a t-test was used, or a nonparametric Mann-Whitney U-test. In all analyses, a 95% confidence level (p < 0.05) was taken as the criterion of significance.

## Results

**Figure 1:**
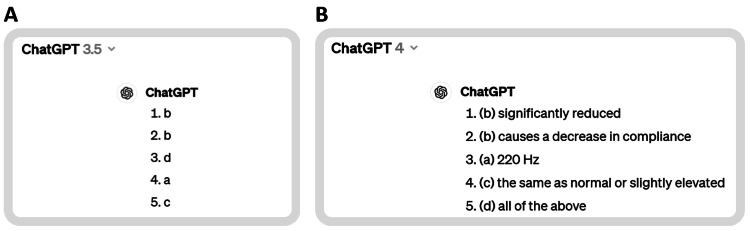
Snapshots of one of the ChatGPT 3.5 (A) and ChatGPT 4 (B) trials. Answers to the first five questions. All answers can be found in the Appendices.

A snapshot of one sample of ChatGPT 3.5 and ChatGPT 4 responses to the first five questions is shown in Figure 1.

**Figure 2:**
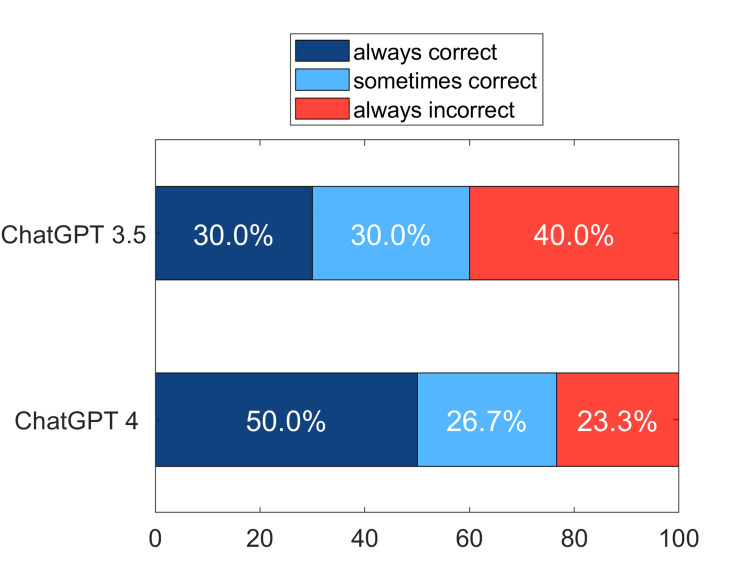
Percentage of 30 questions given correct responses across all 20 trials for two versions of ChatGPT. The bars show percent of questions that were answered correctly on all trials (dark blue), the percent that were sometimes answered correctly and sometimes incorrectly (light blue), and those that were always answered incorrectly (pink).

First the percentage of questions receiving a correct answer on all 20 trials for both versions of ChatGPT was evaluated (Figure 2). For ChatGPT 3.5 the percent of questions that received correct answers at all trials was 30%, while for ChatGPT 4 the corresponding figure was 50%. There was also a fraction of questions that received varied responses, sometimes receiving a correct answer and sometimes an incorrect one. There were also questions that were always answered incorrectly.

The average accuracy of responses (i.e. correctness in relation to the response key) was 48-49% for ChatGPT 3.5 and 65-69% for ChatGPT 4 (Table 2). When looking at minimum and maximum agreement (bottom row of Table 2) there is quite a large spread in the results. Version 3.5 has a variability of 40-60% across all trials, while version 4 has 60-76.7%. rmANOVA of percent agreement results revealed a significant effect of ChatGPT version (F(1,8) = 152.1, p < 0.001), but no effect of day or interaction of day and ChatGPT version.

**Table 2:** Percent accuracy (correctness) of two versions of ChatGPT responses on each testing day in relation to the response key. Mean and standard deviation (in brackets) of five trials at each day provided. Minimums and maximums also provided.

	Week at which test was done	Week 1		Week 2	
	Day at which test was done	Day 1	Day 2	Day 3	Day 4
ChatGPT 3.5	Mean percent agreement	48.7 (6.5)	48.0 (3.8)	48.7 (6.5)	48.0 (6.5)
	Percent agreement Min–max	43.3–60.0	43.3–53.3	40.0–56.7	40.0–56.7
ChatGPT 4	Mean percent agreement	67.3 (4.3)	65.3 (6.5)	66.0 (3.7)	68.7 (5.1)
	Percent agreement Min–max	63.3–73.3	60.0-76.7	63.3-70.0	63.3-76.7

The criterion for a pass for the exams from which the questions were derived was 65%. ChatGPT 3.5 did not reach this level even once, with its highest score being 60%. It did manage to surpass the 50% criterion in 25% of trials (five trials out of 20). On the other hand, ChatGPT 4 surpassed the 65% criterion in 50% of trials (10 trials out of 20).

Next, we analyzed test-retest repeatability. Each single set of responses was compared with the other sets from the same day or different day, irrespective of their correctness.

The repeatability of two versions of ChatGPT within the same day is given in Table 3. It can be seen that the average percent agreement within one day was 76-79% for ChatGPT 3.5 and 87-88% for ChatGPT 4. rmANOVA revealed a significant effect of ChatGPT version (F(1,18) = 57.2, p < 0.001), but no effect of day or interaction of day and ChatGPT version. Cohen’s Kappa had average values of 0.67-0.71 for ChatGPT 3.5 and 0.81-0.84 for ChatGPT 4, meaning substantial agreement for version 3.5 and almost perfect for version 4. In all cases Cohen’s Kappa had p<0.0001, indicating that the observed agreement was not accidental.

**Table 3:** Average repeatability of two versions of ChatGPT responses at each testing day. Percent agreement and Cohen’s Kappa calculated between responses (without taking into consideration accuracy of the responses). Mean and standard deviation (in brackets) provided. In all cases Cohen’s Kappa had p<0.0001, indicating that observed agreement was not accidental.

	Week at which test was done	Week 1		Week 2	
	Day at which test was done	Day 1	Day 2	Day 3	Day 4
ChatGPT 3.5	Percent agreement	76.7 (7.0)	78.0 (5.5)	79.3 (4.4)	77.7 (3.9)
	Cohen’s Kappa	0.67 (0.10)	0.69 (0.08)	0.71 (0.06)	0.69 (0.05)
ChatGPT 4	Percent agreement	86.7 (3.5)	88.0 (6.1)	86.0 (3.4)	86.7 (6.7)
	Cohen’s Kappa	0.82 (0.05)	0.84 (0.08)	0.81 (0.05)	0.82 (0.09)

The repeatability of the two versions of ChatGPT between different days is given in Table 4. It can be seen that the percent agreement between different days was 75-79% for ChatGPT 3.5 and 85-88% for ChatGPT 4. rmANOVA revealed significant effect of ChatGPT version (F(1,48) = 75.8, p < 0.001), but no effect of day or interaction of day and ChatGPT version. For trials within the same day, average Cohen’s Kappa was similar in both versions of ChatGPT. In all cases Cohen’s Kappa had p<0.0001, indicating that the observed agreement was not accidental.

**Table 4:** Average repeatability of two versions of ChatGPT responses between each of the test days. Percent agreement and Cohen’s Kappa calculated between responses (without taking into consideration accuracy of the responses). Mean and standard deviation (in brackets) provided. In all cases Cohen’s Kappa had p<0.0001, indicating that observed agreement was not accidental.

	Time between test sessions	1 day	1 day	1 week	1 week
	Days at which test was done	Day 1 vs Day 2	Day 3 vs Day 4	Day 1 vs Day 3	Day 2 vs Day 4
ChatGPT 3.5	Percent agreement	77.9 (5.2)	78.9 (7.1)	75.1 (7.3)	78.0 (5.7)
	Cohen’s Kappa	0.69 (0.07)	0.71 (0.10)	0.65 (0.10)	0.69 (0.08)
ChatGPT 4	Percent agreement	88.5 (4.6)	84.8 (6.7)	85.1 (4.5)	86.3 (4.3)
	Cohen’s Kappa	0.85 (0.06)	0.80 (0.09)	0.80 (0.06)	0.82 (0.06)

## Discussion

None of the ChatGPT versions we tested provided satisfactory performance regarding questions on auditory physiology. Nonetheless, ChatGPT 4 surpassed ChatGPT 3.5 in both accuracy and consistency over time, demonstrating a 20% improvement in accuracy and a 10% enhancement in consistency. Notably, ChatGPT 3.5 failed to gain a pass mark on any of the 20 trials, while ChatGPT 4 achieved a 50% pass mark. Despite these differences, the response variability remained consistent across different time intervals - minutes, days, and a week. We observed that responses were either consistently correct, consistently incorrect, or variable. For ChatGPT 4, 50% of responses were consistently correct, compared to 30% for ChatGPT 3.5. A notable example of an incorrect response is that for question 3, which asked for the frequency of the measuring tone in tympanometry for a child aged three months. While the typical frequency for adults is 226 Hz (and that was known by ChatGPT), it is well established that for children up to six months of age the test frequency should be 1000 Hz [21,22]. This has been known for several years and is basic information in contemporary audiology, so it is hard to know why ChatGPT made this mistake.

Both versions of ChatGPT occasionally deviated from our instruction to provide only a letter corresponding to the correct answer, sometimes elaborating with a full sentence. It also happened that ChatGPT did not respond for several minutes. We experienced this only in version 4. This idiosyncrasy and instances of delayed responses, particularly with ChatGPT 4, underscore the chatbot's limitations in strictly adhering to instructions.

When our results are placed alongside studies from other disciplines that have examined ChatGPT’s performance on single-answer and multiple-choice questions, we observe a similar trend - a roughly 20% performance improvement with ChatGPT 4 over ChatGPT 3.5 [6,23-25]. Studies in otolaryngology, closely related to audiology, have also shown lower performance scores for ChatGPT 3.5, supporting our findings of improved outcomes with the newer version [26,27].

A separate concern with ChatGPT, and potentially other chatbots, lies in their reliability. Accuracy can vary significantly based on the topic and the complexity of the questions [28]. For instance, studies have shown that the accuracy of ChatGPT 3.5 can range from as high as 70% [6] to as low as 43% [27]. A critical question then arises: are the responses consistent across different trials at various times? Interestingly, the variability in responses appears to be fairly independent of their correctness. For example, as illustrated in Figure 2, although the percentage of correct responses differs by 20% between versions 3.5 and 4, the percentage of changeable responses is relatively similar - 30% for version 3.5 and 26.7% for version 4. These variations in response are important as they might significantly affect an outcome. For instance, with ChatGPT 4, the chance of passing an exam might be as low as 50% or as high as 77%, depending on when the system was queried. This variability underscores the importance of understanding and possibly mitigating inconsistencies so as to enhance the reliability of a particular chatbot application.

Despite the chatbots achieving reasonable accuracy in some areas, their performance is not good enough for professional use or patient support, where nearly perfect accuracy is called for. This study aimed to test repeatability using a set of standardized questions; however, we observed significant errors and variability in responses. This is particularly concerning when ChatGPT generates a narrative or answers an open-ended question without providing reliable sources or, in some cases, citing non-existent references [10]. The ability to track sources and verify responses is invaluable, particularly if responses can vary.

Our findings highlight the critical importance of repeatability as well as accuracy. Even if responses are sometimes correct, this is not enough, especially when users do not have the means to verify what is provided, perhaps leading them to be misled. Our study casts a broader light on the utility of ChatGPT and similar AI-powered chatbots, underscoring their considerable variability in responses, which is a significant limitation. Such variability could pose serious challenges in a clinical setting, misleading patients who rely on receiving correct answers.

Previous studies have indicated that ChatGPT and similar chatbots could play useful roles in a number of different healthcare settings, including hearing [29]. Potential applications for clinicians include generating reports, creating document templates, aiding clinical decision-making, simplifying complex information for patient communication, and supporting education [30]. However, in order to fully realize these capabilities, improvements in ChatGPT's reliability are necessary.

## Conclusions

ChatGPT 4 consistently outperforms ChatGPT 3.5 in terms of accuracy and repeatability over time. However, neither version reached satisfactory standards in answering questions related to auditory physiology, highlighting a significant limitation for their use in specialized fields. This study further contributes to a discussion on the repeatability of ChatGPT responses, underlining that repeatability is as vital as accuracy. The observed high variability in responses from time to time raises concerns about the suitability of ChatGPT for professional applications. It suggests there is a need for improvements to enhance the precision and consistency of these AI systems before they can be reliably integrated into professional settings.

## Appendices

Questions used to test ChatGPT:

Please provide responses to the following questions, giving only the question number and letter of the appropriate answer.

1. The middle ear with advanced otosclerosis shows compliance that is:

(a) normal

b) significantly reduced

(c) slightly elevated

(d) slightly decreased.

2. A disruption of the ossicular chain:

(a) causes an increase in compliance

(b) causes a decrease in compliance

(c) increases pressure in the eardrum cavity

(d) does not change the compliance of the middle ear.

3. The frequency of the measuring tone for tympanometry in a child aged 3 months should be:

(a) 220 Hz

(b) 1000 Hz

(c) 50 Hz

(d) 226 Hz.

4. In a patient with recruitment and sensorineural hearing loss of 50 dB HL, the threshold of the ipsilateral stapedius reflex is:

(a) much lowered

(b) slightly lowered

(c) the same as normal or slightly elevated

(d) no reflex at all.

5. If an ear with normal hearing sensitivity is stimulated and the middle ear muscle reflex is absent contralaterally, then it means that there is:

(a) retrocochlear damage

(b) central facial nerve palsy on the side of the ear where the probe is located

(c) conductive damage in the ear where the probe was placed

(d) all of the above.

6. Which of the following factors affects the amplitude of the click evoked otoacoustic emission signal:

(a) the condition of the auditory nerve

(b) the condition of the outer hair cells

(c) the state of the inner hair cells

(d) the state of the auditory cortex.

7. In sensorineural hearing loss, the amplitude of otoacoustic emissions is:

(a) the same as the norm

(b) greater than normal

(c) less than normal

(d) variable over time.

8. A DP-gram is:

(a) a graph of hearing thresholds as a function of frequency

(b) a graph of tinnitus amplitude as a function of frequency

(c) a plot of the amplitude of distortion product otoacoustic emissions as a function of frequency

(d) a plot of the amplitude of primary tones as a function of frequency.

9. Wave V of auditory brainstem responses is generated by:

(a) the dorsal cochlear nuclei

(b) nuclei of the superior olive complex

(c) nuclei of the lateral lemniscus

(d) inferior thalamus.

10. Wave III of auditory brainstem responses is generated by:

(a) dorsal cochlear nuclei

(b) nuclei of the superior olive complex

(c) nuclei of the lateral lemniscus

(d) inferior thalamus.

11. Wave I of auditory brainstem responses is generated by:

(a) dorsal cochlear nuclei

(b) nuclei of the superior olive complex

(c) nuclei of the lateral lemniscus

(d) the auditory nerve.

12. An auditory brainstem response evoked by a tone pip of 500 Hz at 100 dB nHL represents cochlear activity:

(a) in the entire cochlea

(b) in the basal turn

(c) in the apex

(d) in the middle

13. The average error of hearing threshold determination in auditory brainstem response testing is:

(a) 0 dB

(b) 10 dB

(c) 30 dB

(d) 50 dB.

14. In which of the following types of audiograms does the auditory brainstem response at 500 Hz have the highest frequency specificity for high intensities?

(a) normal

(b) rising

(c) sloping

(d) flat.

15. In conductive hearing disorders, a graph of the latency-intensity function is:

(a) shifted upward with respect to the norm

(b) shifted downward with respect to the norm

(c) shifted to the right with respect to the norm

(d) has the same course as in the norm.

16. In retrocochlear hearing disorders, a graph of the latency-intensity function has the following course:

(a) has the same waveform as in the norm

(b) is shifted upward with respect to the norm

(c) is shifted to the right with respect to the norm

(d) is shifted downward with respect to the norm.

17. For a sensorineural loss of 60 dB in the frequency range of 2000-4000 Hz, a plot of the latency-intensity function for a click stimulus has the following characteristics:

(a) it is shifted to the right

(b) it is steeper

(c) it is shifted upward

(d) it is shifted downward.

18. A prolonged value of interval I-III of the auditory brainstem response indicates:

(a) damage to outer hair cells

(b) prolonged conduction time in the auditory nerve

(c) damage to the inner hair cells

(d) damage to the cochlear nuclei.

19. A prolonged value of the III-V interval of auditory brainstem responses indicates:

(a) sensorineural hearing impairment

(b) brainstem conduction disorder

(c) conductive type hearing impairment

(d) auditory nerve damage.

20. Which of the following statements is correct for auditory neuropathy:

(a) the results of all objective tests of hearing are normal

(b) the results of all objective tests are abnormal

(c) tympanogram is normal, no stapedius reflex, no otoacoustic emissions, correct auditory brainstem response responses

(d) tympanogram is normal, no stapedius reflex, otoacoustic emissions is normal, incorrect auditory brainstem response recording.

21. Which of the following statements is correct when examining an ear with a conductive hearing loss:

(a) impedance audiometry results are abnormal, while otoacoustic emissions and auditory brainstem responses are normal

(b) tympanogram is abnormal, stapedius reflex threshold is normal, otoacoustic emissions are normal, slightly prolonged latencies in auditory brainstem response recordings

(c) tympanogram is abnormal, the stapedius reflex threshold is elevated or absent, no otoacoustic emissions, prolonged latencies in auditory brainstem response recordings

(d) the tympanogram is abnormal, the stapedius reflex threshold is normal, no otoacoustic emissions, prolonged latencies in auditory brainstem response recordings

22. A type C tympanogram indicates:

(a) secretory otitis media

(b) Eustachian tube dysfunction

(c) otosclerosis

(d) interruption of the ossicular chain.

23. A type B tympanogram indicates:

(a) secretory otitis media

(b) Eustachian tube dysfunction

(c) otosclerosis

(d) interruption of the ossicular chain.

24. A type As tympanogram indicates:

(a) secretory otitis media

(b) Eustachian tube dysfunction

(c) otosclerosis

(d) disruption of the ossicular chain

25. A middle ear susceptibility of 0.8 ml indicates:

(a) otosclerosis

(b) interruption of the ossicular chain

(c) secretory otitis media

(d) normal middle ear.

26. A middle ear susceptibility of 0.2 ml suggests:

(a) otosclerosis

(b) interruption of the ossicular chain

(c) secretory otitis media

(d) a properly functioning middle ear.

27. A middle ear compliance of 3.5 ml indicates:

(a) otosclerosis

(b) interruption of the ossicular chain

(c) secretory otitis media

(d) normal middle ear.

28. Which of the following factors does not affect the otoacoustic emission signal?

(a) condition of the outer hair cells

(b) condition of the middle ear

(c) condition of the auditory nerve

(d) the way the probe is placed in the external auditory canal.

29. Which of the following sentences is true:

(a) otoacoustic emissions are almost always present with an A-type tympanogram

(b) otoacoustic emissions are almost always present with a tympanogram type As

(c) otoacoustic emissions are almost always present with a type B tympanogram

(d) otoacoustic emissions are almost always present with a type Ad tympanogram.

30. Auditory brainstem responses have the following clinical applications:

(a) for newborn hearing screening

(b) for hearing threshold testing

(c) for hearing threshold testing, newborn hearing screening, and differential diagnosis of hearing disorders

(d) only for the diagnosis of retrocochlear disorders.

Response key:

1.       b

2.       a

3.       b

4.       c

5.       c

6.       b

7.       c

8.       c

9.       c

10.   a

11.   d

12.   b

13.   b

14.   c

15.   c

16.   b

17.   b

18.   b

19.   b

20.   d

21.   c

22.   b

23.   a

24.   c

25.   d

26.   a

27.   b

28.   c

29.   a

30.   c

**Table 5:** ChatGPT 3.5 responses

	Week 1										Week 2									
	Day 1 (trial number)					Day 2 (trial number)					Day 3 (trial number)					Day 4 (trial number)				
Question number	1	2	3	4	5	1	2	3	4	5	1	2	3	4	5	1	2	3	4	5
1	b	b	b	b	b	b	b	b	b	b	b	b	b	b	b	b	b	b	b	b
2	b	b	b	b	b	b	b	b	b	b	b	b	b	b	b	b	b	b	b	b
3	d	d	d	d	d	d	d	d	d	d	d	d	d	d	d	d	d	d	d	d
4	a	a	a	a	c	a	a	a	a	b	b	a	a	a	b	a	a	a	a	c
5	c	d	d	d	d	d	d	c	d	a	c	d	d	b	c	d	d	d	b	c
6	b	b	b	b	b	b	b	b	b	b	b	b	b	b	b	b	b	b	b	b
7	c	c	c	c	c	c	c	c	c	c	c	c	c	c	c	c	c	c	c	c
8	c	c	c	c	c	c	c	c	c	c	c	c	c	c	c	c	c	c	c	c
9	b	b	d	d	d	a	d	d	b	b	d	b	d	d	b	d	d	d	b	d
10	b	b	b	b	b	b	a	b	a	b	a	a	a	b	a	b	a	b	a	b
11	d	d	d	d	d	d	d	d	d	d	d	d	d	d	d	d	d	d	d	d
12	b	c	b	b	b	b	b	b	b	b	c	c	b	c	b	c	b	c	b	b
13	b	b	c	c	b	b	b	c	c	c	b	c	c	c	c	c	c	c	c	b
14	c	c	b	b	d	b	d	c	b	b	c	c	b	b	c	b	b	c	c	c
15	c	c	a	b	b	c	b	c	c	c	c	c	c	c	c	a	c	c	a	a
16	c	c	c	b	c	c	c	c	c	c	c	c	c	c	b	b	c	c	b	b
17	d	d	d	a	d	a	c	d	c	a	c	d	c	a	a	a	d	c	c	c
18	b	b	b	b	b	b	b	b	b	b	b	b	b	b	b	b	b	b	b	b
19	b	b	b	b	b	b	b	b	b	b	b	b	b	b	b	b	b	b	b	b
20	c	c	c	c	c	c	c	c	c	c	c	c	c	c	c	c	c	c	c	c
21	c	c	c	c	c	c	c	c	c	c	c	c	c	c	c	c	c	c	c	c
22	b	b	b	b	b	b	b	b	b	b	b	b	b	b	b	b	b	b	b	b
23	a	a	a	a	a	a	a	a	a	a	a	a	a	a	a	a	a	a	a	a
24	d	d	d	d	d	d	d	d	d	a	d	d	d	d	d	d	d	a	d	d
25	d	c	d	b	b	c	a	d	d	c	c	c	b	c	b	c	d	c	c	a
26	a	d	d	d	d	d	d	d	d	d	d	d	d	d	d	d	d	d	d	d
27	a	a	c	c	a	a	a	d	a	a	a	a	a	a	a	a	a	a	a	a
28	b	b	b	b	b	b	b	b	b	b	b	b	b	b	b	b	b	b	b	b
29	b	b	b	a	b	b	b	b	b	b	b	b	b	b	c	b	b	b	b	b
30	c	c	c	c	c	c	c	c	c	c	c	c	c	c	c	c	c	c	c	c

**Table 6:** ChatGPT 4 responses

	Week 1										Week 2									
	Day 1 (trial number)					Day 2 (trial number)					Day 3 (trial number)					Day 4 (trial number)				
Question number	1	2	3	4	5	1	2	3	4	5	1	2	3	4	5	1	2	3	4	5
1	b	b	b	b	b	b	b	b	b	b	b	b	b	b	b	b	b	b	b	b
2	b	b	b	b	b	b	b	b	b	b	b	b	b	b	b	b	b	b	b	b
3	b	d	d	a	a	d	a	d	a	a	d	d	d	a	a	d	d	d	d	d
4	c	c	c	c	c	c	c	c	c	c	c	c	c	c	c	c	c	c	c	c
5	a	a	d	a	d	d	d	a	a	d	d	d	a	d	d	a	a	d	a	d
6	b	b	b	b	b	b	b	b	b	b	b	b	b	b	b	b	b	b	b	b
7	c	c	c	c	c	c	c	c	c	c	c	c	c	c	c	c	c	c	c	c
8	c	c	c	c	c	c	c	c	c	c	c	c	c	c	c	c	c	c	c	c
9	b	b	b	b	b	b	b	b	c	b	b	b	c	c	b	b	b	b	c	b
10	a	a	a	a	a	a	a	a	a	a	c	a	a	a	a	a	c	a	a	a
11	d	d	d	d	d	d	d	d	d	d	d	d	d	d	d	d	d	d	d	d
12	c	b	c	c	c	c	c	c	b	c	c	b	c	b	c	c	c	b	c	c
13	b	b	b	b	b	b	b	b	b	b	b	b	b	b	b	b	b	b	b	b
14	d	d	d	d	d	d	d	d	c	d	c	c	d	d	d	c	c	c	d	c
15	a	a	a	a	c	a	a	a	a	a	a	a	a	a	a	a	a	a	c	a
16	b	c	b	c	b	c	b	b	b	c	c	c	c	c	c	c	b	c	b	c
17	a	a	a	a	a	a	a	a	a	a	a	a	a	a	a	a	a	a	a	a
18	b	b	b	b	b	b	b	b	b	b	b	b	b	b	b	b	b	b	b	b
19	b	b	b	b	b	b	b	b	b	b	b	b	b	b	b	b	b	b	b	b
20	d	d	d	d	d	d	d	d	d	d	d	d	d	d	d	d	d	d	d	d
21	c	a	a	a	c	a	a	a	a	a	c	a	a	c	c	a	a	a	c	a
22	b	b	b	b	b	b	b	b	b	b	b	b	b	b	b	b	b	b	b	b
23	a	a	a	a	a	a	a	a	a	a	a	a	a	a	a	a	a	a	a	a
24	c	c	c	c	c	c	c	c	c	c	c	c	c	c	c	c	c	c	c	c
25	d	d	d	d	d	d	d	d	d	d	d	d	d	d	d	d	d	d	d	d
26	a	d	a	a	d	d	d	d	a	a	d	a	d	d	d	a	a	a	a	d
27	d	d	d	d	d	d	d	d	d	d	d	d	d	d	d	d	d	d	d	d
28	c	c	c	c	c	c	c	c	c	c	c	c	c	c	c	c	c	c	c	c
29	a	a	a	a	a	a	a	a	a	a	a	a	a	a	a	a	a	a	a	a
30	c	c	c	c	c	c	c	c	c	c	c	c	c	c	c	c	c	c	c	c
